# A diagnostic LAMP assay for the destructive grapevine insect pest, phylloxera (*Daktulosphaira vitifoliae*)

**DOI:** 10.1038/s41598-020-77928-9

**Published:** 2020-12-04

**Authors:** Arati Agarwal, J. Paul Cunningham, Isabel Valenzuela, Mark J. Blacket

**Affiliations:** 1 Agriculture Victoria Research, AgriBio, 5 Ring Road., Bundoora, VIC 3083 Australia; 2 School of Applied Systems Biology, La Trobe University, Bundoora, VIC 3083 Australia

**Keywords:** High-throughput screening, Molecular biology, Agroecology

## Abstract

Grape phylloxera (*Daktulosphaira vitifoliae*) is a destructive insect pest of grapevines that is highly invasive worldwide, despite strict biosecurity containment measures in place at farm and regional levels. Current phylloxera identification by visual inspection and laboratory-based molecular methods is time-consuming and costly. More rapid and cost-effective methods for identification of this pest would benefit industry, growers, and biosecurity services. Loop mediated isothermal amplification (LAMP) is a new portable technology available for rapid and accurate in-field molecular diagnostics. This study outlines the development of a new LAMP assay to enable the identification of phylloxera specimens. New LAMP primers were developed to specifically amplify phylloxera mitochondrial DNA (5′-COI), which we have shown is effective as a DNA barcode for identification of phylloxera, using LAMP technology. Positive LAMP reactions, containing phylloxera DNA, amplified in less than twelve minutes with an anneal derivative temperature of approximately 79 °C to 80 °C compared to a newly designed synthetic DNA (gBlock) fragment which had an anneal derivative temperature of 82 °C. No LAMP amplification was detected in any of the non-target species tested, i.e. no false-positive identification resulted for these species. We also successfully optimised a non-destructive DNA extraction procedure, HotSHOT “HS6”, for use in the field on phylloxera adults, nymphs and eggs, to retain physical specimens. DNA extracted using this method was also suitable for species and genotype molecular identification methods, such as DNA barcoding, qPCR and microsatellite genotyping. The new LAMP assay provides a novel visual molecular tool for accurate diagnostics of phylloxera in the laboratory and field.

## Introduction

Grape phylloxera (*Daktulosphaira vitifoliae*) is the most economically important insect pest of grapevines^1,2^. Originally native to north America, this tiny aphid-like pest has been accidentally introduced to viticultural regions worldwide^1–4^. Phylloxera directly damage plants through feeding and can induce leaf or root galling, with root feeding causing the greatest damage, which can often ultimately lead to the death of infested vines^1–3^. Control for phylloxera is currently limited to (i) surveillance and quarantine measures aimed at reducing human mediated spread to new sites, and (ii) measures to reduce the impact of established populations^2–4^, including the use of resistant rootstocks, produced through crossing varieties and species of *Vitis*, or application of systemic insecticides, which must be continuously applied.

Outside their native range phylloxera have a high likelihood of establishment once introduced to new areas as they have been found to reproduce through parthenogenetic asexual reproduction when introduced to new regions, such as California, Europe and Australia^4,5^. Clonal strains of phylloxera differ in their life histories and ecological impact on host vines, with individual asexual strains currently recognized through genotyping of multilocus nuclear microsatellites^4,5^.

Phylloxera primarily live underground on the roots of grapevines, where they are very difficult to detect until the symptoms of infestation (stunted growth and premature yellowing of leaves) appear^2^. Existing surveillance practices for phylloxera rely on visual inspection of vines and roots by trained entomologists, particularly those vines showing signs of weakness that may be due to the presence of the insect. Above ground trapping is also used to monitor for mobile lifestages emerging from the soil, which become caught in emergence traps secured on the ground close to vines^6^. Suspect phylloxera detected using either of these methods must then be examined by experts in morphological taxonomy, to confirm whether the specimen is phylloxera; a process that is time consuming (up to a week for diagnostics) and costly. Current laboratory methods for molecular identification, which include DNA barcoding of the 5′ region of the Cytochrome Oxidase I (COI) locus^7,8^, real-time qPCR^9^, and microsatellite genotyping^4^, also involve significant time and monetary costs. More rapid and cost-effective identification methods for phylloxera that could be used directly in the field would therefore be of considerable benefit to biosecurity officers, consultants, the grape and wine industry and research.

LAMP (loop-mediated isothermal amplification) is a relatively new molecular technology^10^ that is now starting to be used for rapid and accurate diagnostics of insect pests^11^. Analysis is fast—LAMP tests can detect DNA based targets in less than an hour—and results are visual and easy to interpret^11^. LAMP amplification can be conducted in robust portable devices that can be applied to field-based (on-site) diagnostic testing, being able to handle less refined “crude” DNA extracts compared to conventional molecular techniques such as PCR.

This study details development and testing of a new LAMP diagnostic test for identification of phylloxera, with the following aims: (i) to demonstrate that DNA sequence variation in the 5′-COI DNA barcoding region can identify grape phylloxera, (ii) to develop and optimise a LAMP assay for the accurate detection of phylloxera using the COI locus, (iii) to test a field-deployable DNA extraction method for obtaining “crude” phylloxera DNA samples, while retaining physical voucher specimens, (iv) to develop protocols suitable for testing phylloxera in the field, (v) to validate the new assay through testing the specificity against the target phylloxera and non-target samples, (vi) to design and evaluate detection sensitivity of a gBlock dsDNA fragment for use as synthetic DNA positive in phylloxera LAMP assays.

## Results

### Molecular variation

There are more than two hundred phylloxera reference 5′-COI DNA barcode sequences, which are up to 4.5% divergent from each other—from the USA, Canada, Netherlands and China—including published^8,12^ and unpublished sequences on BOLD, http://www.boldsystems.org, accessed July 2020. The next genetically most similar species on BOLD and GenBank are > 7.5% divergent from phylloxera. Therefore, phylloxera can be readily identified using DNA barcoding. Australian phylloxera G1 and G4 strains were found to be genetically divergent from each other (3.5%) and from the other five strains examined (1.4 to 3.8%) for the 5′-COI DNA region. The genetic variation present within these Australian strains is representative of general phylloxera diversity present on BOLD, being > 99% similar to reference sequences on BOLD (Supplementary Fig. 1). The genetically most similar non-target insects sequenced and tested using LAMP in our study, *Adelges* spp., were > 9% divergent from phylloxera. The phylloxera and non-target invertebrate DNA barcode sequences obtained in the current study have been submitted to GenBank (MW292598-MW292628).

**Figure 1:**
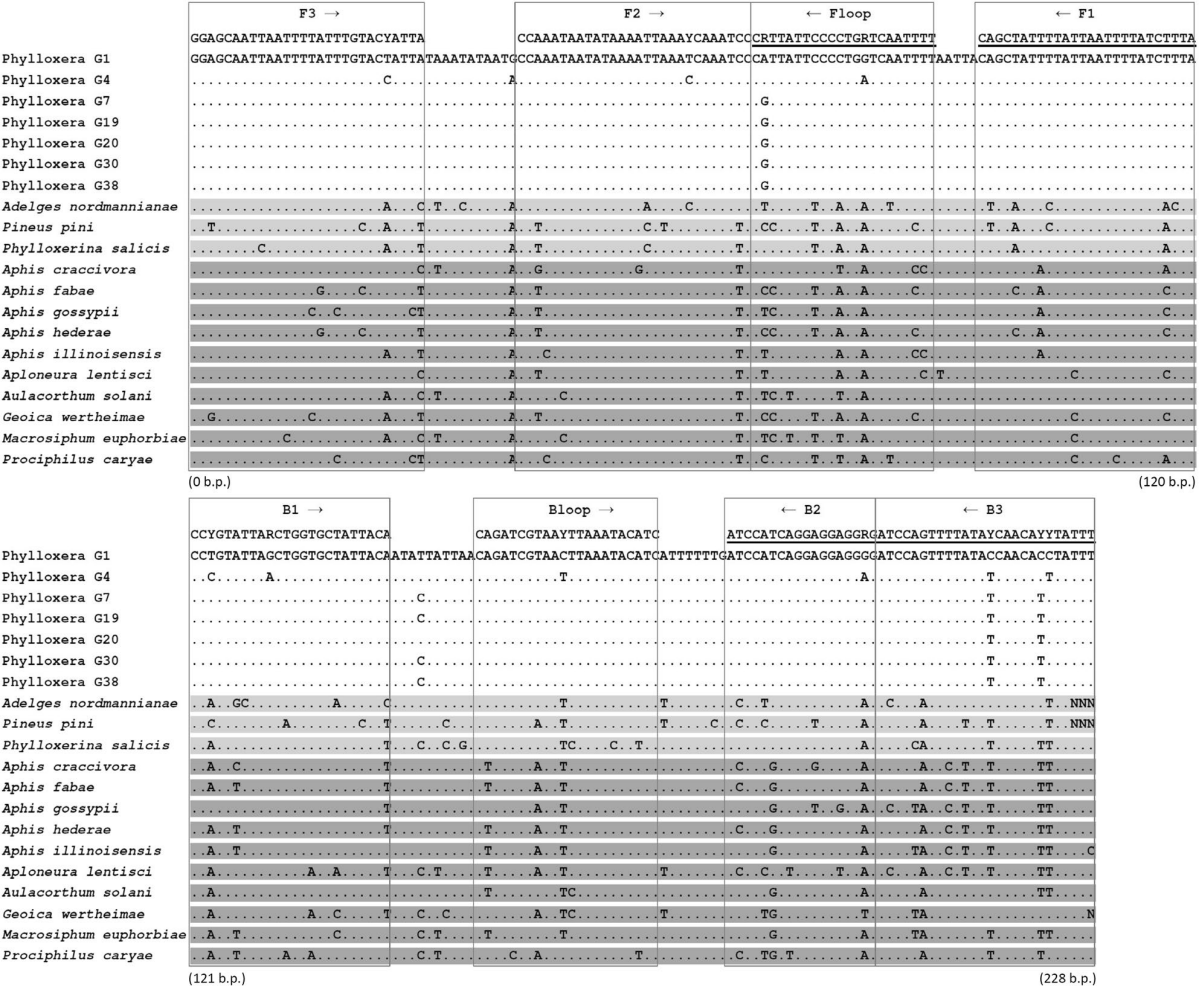
Alignment of COI DNA sequences of phylloxera and related non-target species (see Supplementary Table 1). Boxes indicate the eight primer regions used for designing six primer pairs. Reverse primers are underlined; FIP (5′–3′) is made by combining F1 (reverse compliment) and F2; BIP (5′–3′) is made by combining B1 (reverse compliment) and B2. Sequences of target phylloxera (G1–G38) are non-shaded, other Phylloxeroidea are shaded light grey and Aphidoidea are shaded dark grey.

### LAMP assay design

Six novel LAMP assay primers (Table 1), for eight sections of a contiguous 228 base pair region of the 5′-COI locus (Fig. 1), were designed for detection of phylloxera in the present study. These primers were designed by eye from an alignment which included COI DNA sequences (Fig. 1) from phylloxera and related Aphidomorpha non-target species (Supplementary Table 1). DNA sequences from the seven phylloxera laboratory strains, were used for designing LAMP primers (Fig. 1), with ambiguous DNA bases added to LAMP primers to account for variation observed in DNA barcodes of phylloxera genotypes and reference sequences on BOLD. The phylloxera LAMP assay consists of six primers, the outer forward primer F3, the outer backward primer B3, the inner forward primer FIP, the inner backward primer BIP, the forward loop primer Floop and the backward loop primer Bloop (Fig. 1). The optimised primer ratio (F3/B3: FIP/BIP: Floop/Bloop) for this assay was determined to be 1:6:3, with the final concentrations of 0.4 µM, 2.4 µM and 1.2 µM for the F3/B3, FIP/BIP and Floop/Bloop primers respectively. The use of loop primers resulted in rapid amplification times producing optimum amplification time for target species. A 221 bp gBlock dsDNA fragment (Table 1) was designed for use as synthetic DNA positive control for the phylloxera LAMP assay.

**Table 1:** LAMP primer and amplicon sequences (gBlock) and parameters. The F2 and B2 primer regions of FIP and BIP are underlined.

LAMP primer or amplicon	Sequence 5′–3'	Primer length (bp)	Predicted Tm, annealing temperature (°C)	Degeneracy of primer (fold)
Phylloxera gBlock fragment	cccGGAGCAATTAATTTTATTTGTACTATTAcccCCAAATAATATAAAATTAAATCAAATCCcccCATTATTCCCCTGGTCAATTTTcccCAGCTATTTTATTAATTTTATCTTTAcccCCTGTATTAGCTGGTGCTATTACAcccCAGATCGTAACTTAAATACATCcccATCCATCAGGAGGAGGGGcccATCCAGTTTTATACCAACACCTATTTccc	221	N/A	None
Phy-F3	GGAGCAATTAATTTTATTTGTACYATTA	28	58.2	2
Phy-B3	AAATARRTGTTGRTATAAAACTGGAT	26	59.8	8
Phy-FIP	TAAAGATAAAATTAATAAAATAGCTGCCAAATAATATAAAATTAAATYCAAATCC	55	68.5	2
Phy-BIP	CCYGTATTARCTGGTGCTATTACACYCCTCCTCCTGATGGAT	42	75.3	8
Phy-FL	AAAATTGAYCAGGGGAATAAYG	22	62.2	4
Phy-BL	CAGATCGTAAYTTAAATACATC	22	57.7	2

### LAMP assay results

**Figure 2:**
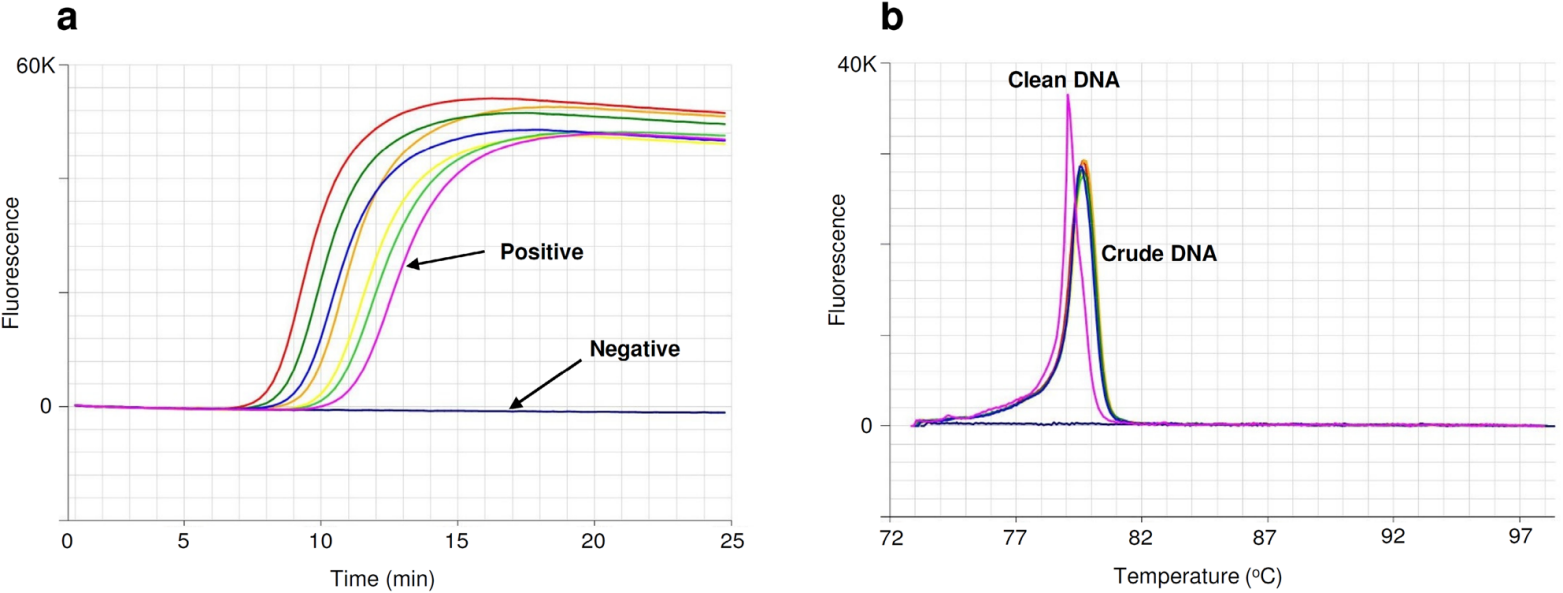
Optimised LAMP assay performed on adult phylloxera using crude and clean DNA extracts. (**a**) Amplification profile, with positive samples amplifying in < 12 min. (**b**) Anneal derivative of LAMP amplicons, with an anneal derivative of approximately 79 °C to 80 °C.

Positive LAMP reactions from phylloxera DNA amplified in less than twelve minutes, with anneal derivative temperatures of approximately 79 °C to 80 °C (Table 2a, Fig. 2) with amplification in less than 25 min considered positive. Amplification was robust to DNA extraction method, with the crude HotSHOT HS6 procedure producing results comparable to the clean Qiagen DNA extractions (G1 to G38) (Table 2a, Fig. 2).

**Table 2:** Performance of the Phylloxera LAMP assay using the optimised primer ratio and concentration (1:6:3; 0.4 µM). (**a**) Phylloxera specimens from different life stages, “crude” DNA (non-destructive) and “clean” DNA (destructive) extractions. (**b**) Phylloxera “crude” DNA (non-destructive) extracts from all life stages, blind panel test (workshop results).

Specimens^†^	n	Amplification time (min)			Anneal derivative temperature (^o^C)		
		Mean	SD	Min–Max	Mean	SD	Min–Max
**a**							
Adult	6	11.0	1.0	10.0–12.8	79.7	0.2	79.5–79.9
Crawler	6	10.5	0.6	10.0–11.5	79.8	0.2	79.6–80.0
Egg	6	10.3	1.0	9.0–11.5	79.7	0.1	79.6–79.7
G1–G38*	7	11.3	0.9	10.5–11.5	79.7	0.2	79.5–79.9
Positive (G4)	3	11.8	0.5	11.3–12.3	79.4	0.3	79.1–79.7
**b**							
Egg	6	12.4	1.0	11.0–14.0	79.5	0.3	79.1–79.8
Crawler	3	13.8	0.4	13.5–14.3	79.5	0.3	79.3–79.8
Intermediate	5	13.5	2.0	12.3–17.0	79.4	0.2	79.2–79.6
Adult	10	12.1	0.6	11.0–13.0	79.3	0.2	79.1–79.6
Alate	1	14.3	0.0	14.3	79.4	0.0	79.4
Positive (G 30)	5	11.5	0.9	10.0–12.3	79.5	0.5	79.1–80.3

^†^Adult, crawler, egg, intermediate, adult and alate “crude” DNA extract (G4 strain) HotSHOT HS6 method. G1–G38, *seven strains G1, G4, G7, G19, G20, G30 and G38 of adult phylloxera “clean” DNA extract, Qiagen kit.

LAMP amplification was not sensitive to the DNA extraction method employed, with “crude” DNA extractions providing consistent results (Table 2b, Fig. 2). A training workshop was held at the Agriculture Victoria Rutherglen Centre, including ten AgVic staff participants who were previously untrained in using LAMP. Phylloxera samples from the laboratory colonies, including all life stages, were used for this workshop. The LAMP assay results generated by the workshop participants produced positive results from all samples (Table 2b).

**Figure 3:**
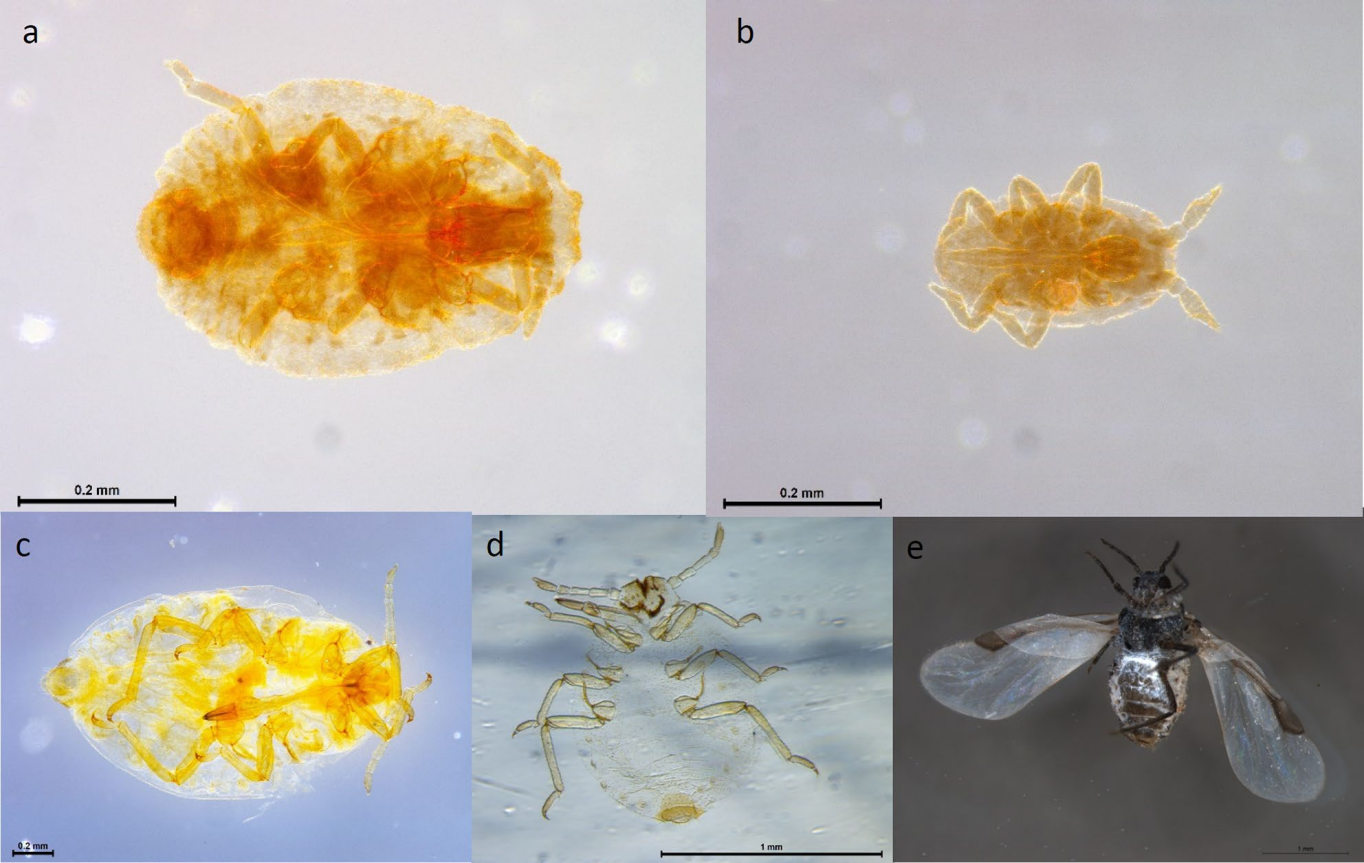
Insect specimens collected from grapevines used for testing the specificity of the LAMP assay, target phylloxera (upper), non-target root aphids (lower). (**a**) Phylloxera adult, post-HotSHOT DNA extraction (**b**) first instar nymph (crawler), post-HotSHOT DNA extraction. (**c**) *Aploneura lentisci* CHS19-001,161. (**d**) *Smynthurodes betae*, VAITC10006. (**e**) *Geoica* sp., VAITC8809.

Additionally, these “crude” DNA extractions could be used for all available molecular methods of phylloxera detection—DNA barcoding, qPCR and microsatellite genotyping (Table 3). Both five microlitres and two microlitres of template DNA used in qPCR performed equally well producing the expected Cq value. Non-destructive HotSHOT HS6 extractions still retained the key characters used for morphological identification of phylloxera (Fig. 3).

**Table 3:** Performance of the phylloxera non-destructive crude DNA samples from three life stages tested for application in LAMP, real-time qPCR, microsatellite genotyping, and DNA barcoding.

Specimens^†^	n	LAMP		qPCR (Cq value)		Microsatellite	DNA barcoding
		Amplification time (min)	Anneal derivative temperature (°C)	5 µl DNA	2 µl DNA		
		Mean ± SD	Mean ± SD	Mean ± SD	Mean ± SD	(genotype)	(match on BOLD)
Egg	3	14.0 ± 2.6	80.5 ± 0.6	22.7 ± 3.5	24.7 ± 2.7	G4	*D. vitifoliae*,100%
Crawler	3	15.8 ± 0.0	79.1 ± 0.1	25.3 ± 1.1	26.3 ± 1.0	G4	*D. vitifoliae*,100%
Adult	3	18.6 ± 3.8	78.7 ± 0.2	23.9 ± 3.6	25.4 ± 3.6	G4	*D. vitifoliae*,100%

^†^G4.

### Validation of the assay

**Figure 4:**
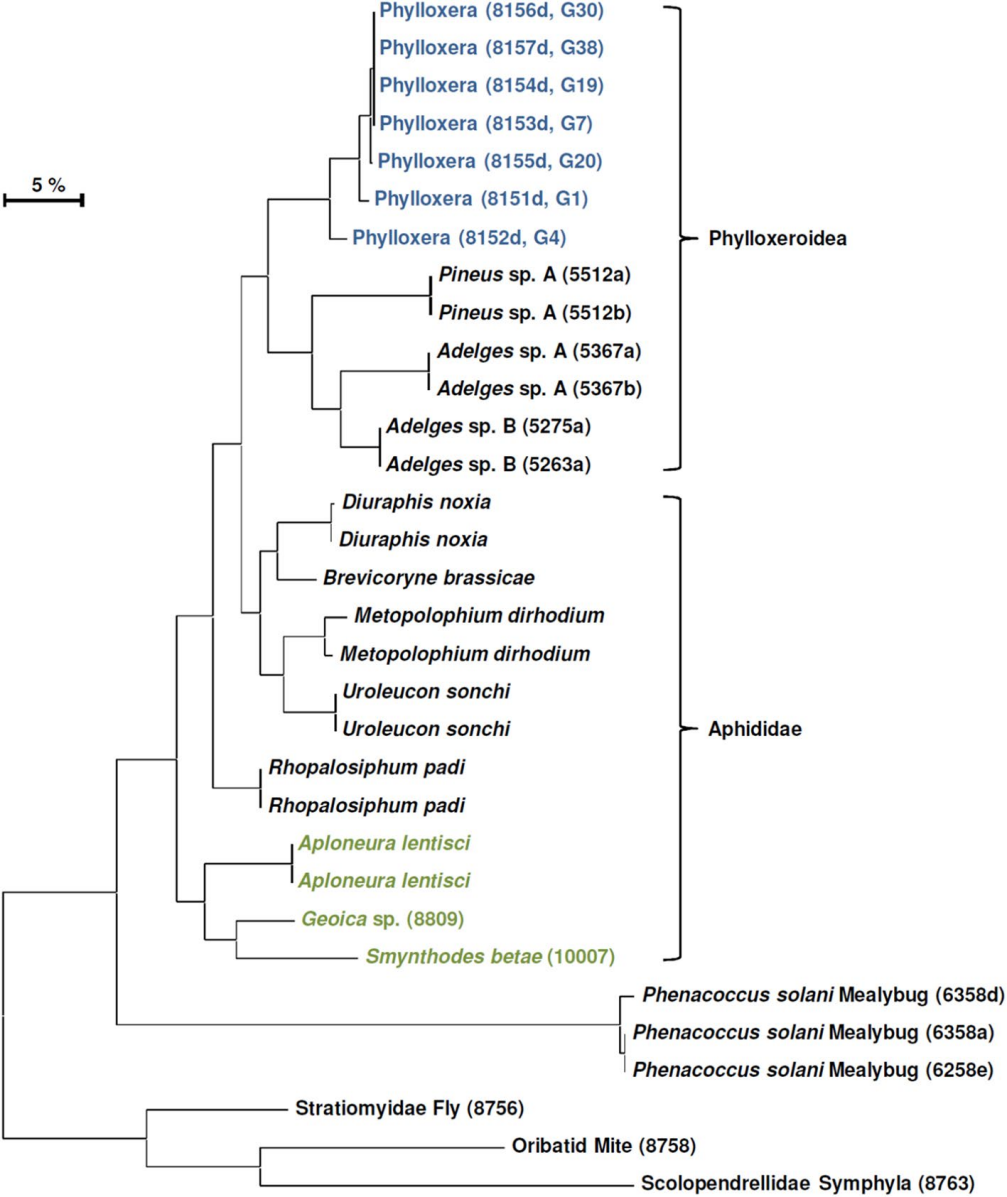
Maximum Likelihood tree of DNA samples used for testing phylloxera LAMP assay. Phylloxera samples in blue, root aphid samples in green, specimen reference collection identification numbers in brackets.

The specificity of the phylloxera LAMP assay was validated against a broad range of non-target taxa (Fig. 4), with no off-target amplification observed. These taxa included many species of Phylloxeroidea and Aphididae (including three species of root aphid), as well as other more distantly related arthropods (Fig. 4).

**Figure 5:**
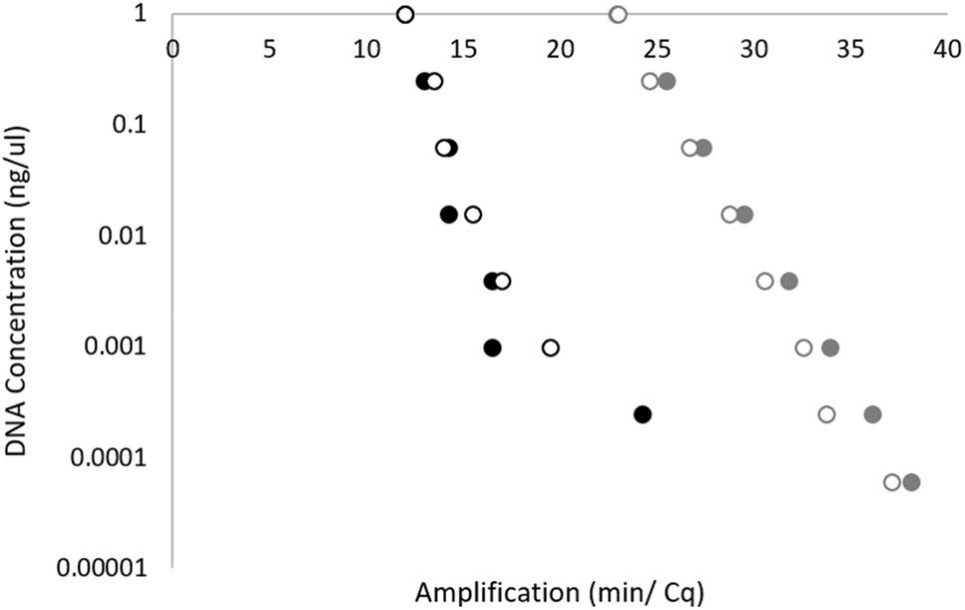
Dilution series comparison of phylloxera LAMP and phylloxera real-time qPCR assays. Black circles, LAMP DNA dilution series amplification times for two biological replicates of phylloxera. Grey circles, Real-time qPCR, DNA dilution series Cq values, DNA samples as above.

The sensitivity of the phylloxera LAMP assay (Fig. 5) and qPCR (Fig. 5) were tested using a fourfold serial dilution of phylloxera DNA. The results from both assays appearing very similar, both proving to be very sensitive. QPCR was more sensitive producing positive results even at the lowest DNA concentration 6.1E−05 ng/µL compared to LAMP which was sensitive to 2.4E−04 ng/µL.

### Detection sensitivity of gBlock DNA fragment

**Figure 6:**
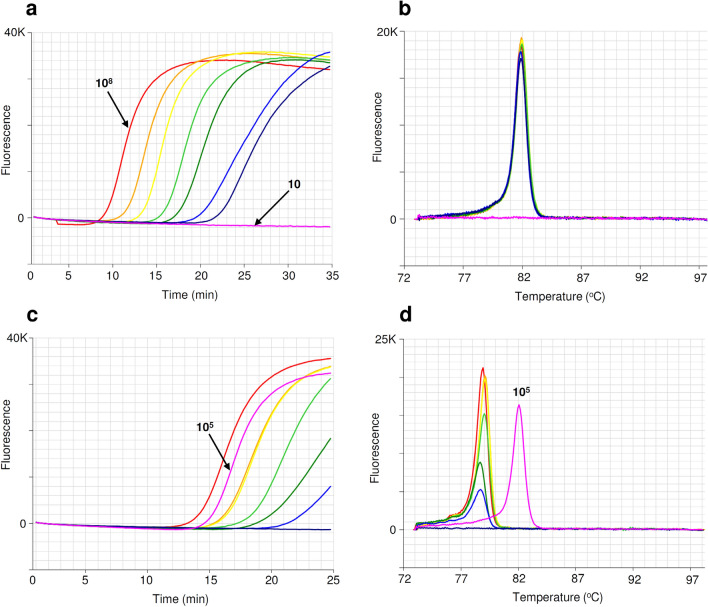
Detection sensitivity of phylloxera gBlock dsDNA amplicons (upper), comparison of phylloxera DNA dilution series with synthetic DNA (lower). (**a**) Amplification profile with templates ranging from 10^8^ to 10 copies at tenfold dilution (pink, no amplification). (**b**) Anneal derivative of LAMP amplicons, with an anneal derivative of 82 °C. (**c**) Amplification profile of fourfold dilution of phylloxera DNA ranging from 1 ng/µL to 0.00098 ng/µL and synthetic DNA (10^5^ copies, pink). (**d**) Anneal derivative of LAMP amplicons with two peaks, 79 °C for phylloxera DNA and 82 °C for synthetic DNA (pink).

The detection sensitivity of phylloxera gBlock dsDNA fragment (Fig. 6) was evaluated for templates ranging from ~ 100 million copies down to ~ 10 copies at tenfold dilution in LAMP reactions. The detection level was quite sensitive, detecting as low as ~ 100 copies within 25 min (Fig. 6a) with an anneal derivative of 82 °C (Fig. 6b). One hundred thousand copies of gBlock fragment was compared to fourfold dilution of phylloxera DNA ranging from 1 ng/µL to 0.00098 ng/µL (Fig. 6c). From the amplification profile one hundred thousand copies of synthetic DNA equates to ~ 0.5 ng/µL of phylloxera DNA. The anneal derivative of LAMP amplicons in this run shows two peaks, 79 °C for phylloxera DNA and 82 °C for synthetic DNA fragment (Fig. 6d).

## Discussion

Effective surveillance for the presence of phylloxera in vineyards is currently challenged by taxonomic identification methods that require significant costs in time and money^4,6^. In our study we have successfully developed and tested a new LAMP assay for the rapid and accurate identification of phylloxera. The assay was demonstrated to be specific for phylloxera with no amplification of non-target taxa observed. We tested the assay on a representative range of phylloxera genotypes from Australia, including the two root galling “superclones”, G1 and G4, which are highly abundant, persistent, geographically widespread in Australia, and are known to cause extensive damage to ungrafted vines^5,13,14^. The range of genetic variation used to design the LAMP assay encompasses the known range of phylloxera 5′-COI variation worldwide, and the new assay should prove useful for the different ecological forms of phylloxera, including both root and leaf galling forms. The new LAMP assay is designed to detect the species *Daktulosphaira vitifoliae* but cannot differentiate between phylloxera strains. Currently, the only method available to differentiate strains is microsatellite genotyping^4^, with multilocus microsatellites not being suitable for development of LAMP assays. Newly characterized genomic information^15^ may provide future strain-specific single-locus molecular markers for use in the identification of phylloxera strains.

In the context of quarantine, and early detection after initial infestation, it is critical that field assays can detect single insects of all lifestages. Our new assay was found to be consistently accurate across all genetic strains tested and on all lifestages, even when DNA was present in very low concentrations (e.g. single egg); and sensitive down to very low DNA levels, similar to the existing qPCR test^9^. However, LAMP is potentially an even better tool for uptake and adoption by biosecurity officers and industry than qPCR, as it is relatively simple to use in the field, with LAMP reagents being capable of amplifying unpurified DNA extractions. LAMP is also a relatively simple procedure requiring limited training (e.g. through a training workshop). “Crude” DNA extractions can be performed in the LAMP machine, with amplification results available in less than one hour.

Field use of the LAMP assay required a simple DNA extraction method that is cheap, rapid and highly reliable. The published HotSHOT protocol has been widely used and is rapid and inexpensive, generating good quality genomic DNA for PCR and genotyping^16^. Genomic DNA can be extracted successfully by briefly incubating the tissue sample in hot sodium hydroxide and pH adjusted with a Tris solution (HotSHOT). Specimens extracted using field compatible non-destructive method, HotSHOT HS6, still retained the key characters used for morphological identification of phylloxera^17^, including segmentation of the antennae, sculpturing of the body as well as relative size of the mouthparts and legs. The use of a non-destructive DNA extraction method for biosecurity is important as morphological vouchers provide physical evidence for the presence of this regulated pest.

In our study we designed and optimised a synthetic DNA positive control (gBlock) for use in phylloxera LAMP assays. This synthetic DNA can be readily identified compared with LAMP amplification from phylloxera DNA through anneal derivative profiles (> 2 °C difference). The use of synthetic DNA as a positive control is beneficial in: (i) providing a consistent control to allow tracking of the performance of LAMP assays across runs, (ii) providing a relatively high amount of control DNA compared with DNA extractions from phylloxera specimens which produce very low DNA yields due to the tiny size of these insects, and (iii) potential nucleic acid transport issues relating to this being a regulated pest in Australia, with phylloxera requiring tracking using permits when moving between management zones.

The development and optimisation of the new phylloxera LAMP assay in our study, suitable for detection of individual insects, now provides a cost effective, rapid, consistent tool for use for identification in the laboratory or in the field, which compliments currently available methods of identification of this destructive insect pest.

## Materials and methods

### Specimens examined

Seven Australian genetic strains (clonal genotypes) of phylloxera were assessed in this project: G1, G4, G7, G19, G20, G30 and G38; genotype numbers follow^14^. These strains were obtained from live insect colonies maintained by Agriculture Victoria Rutherglen, Victoria. Root aphids, mealybugs and other non-target invertebrates were collected from vineyards for assay specificity testing. Additional aphids, from grass and vegetable host plants, as well as Phylloxeroidea (*Adelges* spp. and *Pineus* sp.) from pine trees, were also collected to create a comprehensive Aphidomorpha (Phylloxeroidea/Aphidoidea) species panel to test the specificity of the new LAMP assay.

### Phylloxera DNA extractions and molecular variation

DNA was extracted from 100% ethanol preserved whole phylloxera adult, crawler, and eggs (destructive extraction method) using a DNeasy Blood and Tissue extraction kit (Qiagen, Australia), following the manufacturers protocol. The DNA concentrations were quantified using a Qubit 2.0 Flourometer (Invitrogen, Life Technologies, Australia) following the manufacturers protocol and stored at − 20 °C. These samples provided “clean” DNA preparations to use for phylloxera DNA barcoding, to assess Cytochrome Oxidase I (COI) genetic variation between strains, and for development of the LAMP assay.

All specimens, both phylloxera and the non-target invertebrates, were identified morphologically, prior to DNA extraction, followed by molecular identification using standard DNA barcoding methods^7,17^. DNA sequences from the 5′ region of the COI locus were obtained using LCO1490 / HCO2198 primers^19^. The thermal cycling conditions consisted of a PCR amplification profile of: 2 min at 94 °C, 40 cycles of at 94 °C for 30 s, 50 °C for 45 s and 72 °C for 45 s, and a final extension of 2 min at 72 °C.

PCR amplicons were sanger sequenced by Macrogen Inc. (Seoul, Korea) and sequences were compared to public databases (NCBI and BOLD), to determine similarity (i.e. > 99% matches) with previously identified reference species. DNA sequences from the laboratory strains of phylloxera were used to develop phylloxera specific LAMP primers in this research.

An additional in-field compatible non-destructive DNA extraction method for “crude” DNA extractions was tested. Intact specimens of phylloxera (egg, crawler, and adult) were processed using a modified HotSHOT protocol “HS6” from^20^. There are several variations of HotSHOT method which have been used previously to extract DNA from mice (HS4)^16^ and Zebrafish (HS3)^21^. The HotSHOT (HS6) protocol has been refined to further improve the efficiency of extracted DNA by combining NaOH and Tris–EDTA buffer in a single step^20^. In our study twenty microlitres of HotSHOT extraction buffer consisting of 25 mM NaOH + TE buffer, pH 8.0 (Invitrogen , Australia) (1:1 volume) was pipetted into each 8 well strip of LAMP PCR tubes (OptiGene, UK). Phylloxera samples were removed from ethanol and air dried on a paper towel for approx.1 min. Single whole adult, crawler, single and/or multiple eggs were transferred with a toothpick (single use to prevent cross contamination), into each well. Each sample was immersed in HotSHOT buffer with up to six samples processed simultaneously in the portable real-time fluorometer (Genie III, OptiGene, UK). The protocol used the Genie III as an incubator at 95 °C for 5 min followed by > 1 min incubation on ice. The DNA was quantified using a NanoDrop ND-1000 Spectrophotometer (Thermo Fisher, Australia) and stored at − 20 °C.

### Development of the LAMP assay

#### LAMP primer design

Phylloxera specific primers were designed from an alignment of publicly available COI (5′-region) DNA sequences (from BOLD, http://www.boldsystems.org), which included (i) target (phylloxera) and (ii) non-target species that were either closely related to phylloxera (i.e. Phylloxeroidea), or aphids (Aphididae) known to utilise grapevines or plant roots as hosts (Supplementary Table 1).

For all primers, the GC content (%), predicted melting temperature (Tm), and potential secondary structure (hairpins or dimers) were analysed using the Integrated DNA Technologies (IDT) online OligoAnalyzer tool (https://sg.idtdna.com/calc/analyzer), using the qPCR parameter sets. Complete sets of LAMP primers were analysed together to detect potential primer dimer interactions using the Thermo Fisher Multiple Primer Analyzer tool (www.thermofisher.com). Potential within-species genetic variation in phylloxera LAMP primers was examined using combined DNA sequences of Australian phylloxera and reference sequences available on BOLD (i.e. the seven laboratory strains, plus 230 × 5′-COI reference sequences on BOLD, accessed May 2018).

A synthetic 221 bp DNA fragment, designed from the complete fragment used for the assay (Fig. 1), was synthesized as a gBlock (Integrated DNA Technologies, Iowa, USA) to provide a reliable LAMP positive control. This fragment consisted of all eight LAMP primer regions (Fig. 1) concatenated together, with the non-primer DNA removed from between primers and replaced a run of “ccc” DNA bases between the primer regions and at the ends of the fragment, to increase the overall GC content of the synthetic fragment.

#### LAMP primer ratio optimisation

Primer master mix was prepared following published protocols^11^. Optimisation of the six primer (Table 1) ratio and concentration for the phylloxera LAMP assay was also conducted according to^11^.

#### LAMP assay conditions

The phylloxera LAMP assay was performed following the same method as published^11^. All LAMP assays were run in the Genie III at 65 °C for 25 min followed by an annealing curve analysis from 98 °C to 73 °C with ramping at 0.05 °C/s. The total run time being approximately 35 min.

### Comparison of molecular methods for phylloxera identification

#### Analytical sensitivity of the LAMP assay compared with qPCR

Clean DNA was extracted from phylloxera (3 adult samples were pooled for one DNA extraction) using the destructive method. A fourfold serial dilution of two biological replicates was prepared using ultrapure water. Starting DNA concentrations were quantified using a Qubit 2.0 Flourometer (Invitrogen, Life Technologies, Australia) following the manufacturers protocol. Phylloxera DNA was serially diluted from 1.0 ng/µL to 0.000061 ng/µL (1:1 to 1:16,384). Sensitivity of the LAMP assay was tested using the serially diluted DNA in the Genie III, following the same assay conditions as mentioned above. The time of amplification and anneal derivative temperature was recorded for all samples.

The same serial dilution of DNA extracts was also used in real-time qPCR assay. The primers and probe set (manufactured by Sigma, Australia) and cycling conditions used were as published^9^. Real-time qPCR was performed in QuantStudio 3 Real time PCR system (Thermo Fisher Scientific, Australia) in a total volume of 25 µL with technical replicates for each dilution. Each reaction mixture included 12.5 µL Platinum Quantitative PCR SuperMix-UDG (Invitrogen, Australia), 0.5 µM of each forward and reverse primers, 0.2 µM Taqman probe, 4 µL of template DNA and made up to 25 µL with RNA-free water. An NTC with 4 µl of water instead of DNA was included in each run to check for reagent contamination. The thermal cycling conditions consisted of a two-step denaturation: 2 min at 50 °C and 10 min at 95 °C, followed by 40 cycles of amplification in a two-step procedure: 95 °C for 15 s and 60 °C for 1 min. The mean Cq value (cycling quantification value) of the 8 dilutions were recorded for comparison with the time of amplification obtained from the LAMP assay.

#### Performance of non-destructive DNA samples (HotSHOT HS6)

DNA was non-destructively extracted from whole phylloxera egg, crawler, and adult (n = 3) using the “crude” HotSHOT HS6 protocol, as mentioned above. The extracted DNA was tested across the full range of molecular techniques available for detection of phylloxera: real-time qPCR (using 5 µL and 2 µL DNA per reaction)^9^, microsatellite genotyping^14^, DNA barcoding^7^, as well as the new LAMP assay. Real-time qPCR and DNA barcoding were performed as described above. Microsatellite genotyping was performed following a laboratory protocol (Blacket et al*.* unpublished) that screens the six phylloxera microsatellite loci used to define Australian genotypes^5,14^, modified to utilise fluorescently labelled primer tails following^22^ and a multiplex PCR kit (Qiagen, Australia). Capillary separation of fluorescently labelled microsatellites was performed commercially by AGRF (Melbourne), using LIZ-500 size standards. Genotyping of fsa files was performed using the microsatellite plugin in Geneious R11 (https://www.geneious.com).

Phylloxera specimens (adults and crawlers) were visually examined pre-DNA extraction, with slides prepared from specimens’ post-DNA extraction to confirm retention of key morphological characters. Microscopic slides were prepared following the protocols as published^22^ and were deposited as reference specimens in the Victorian Agricultural Insect Collection (VAIC) (https://collections.ala.org.au).

### Evaluation of gBlock DNA fragment for use as synthetic DNA positive in LAMP assay

To evaluate detection sensitivity a tenfold serial dilution of the gBlock DNA fragment was prepared using ultrapure water (Invitrogen, Australia). Synthetic DNA was serially diluted from ~ 100 million copies down to ~ 10 copies (10^8^ copies to 10 copies). Sensitivity of the LAMP assay was tested using the serially diluted synthetic DNA in the Genie III, following the same assay conditions as mentioned above (run time increased from 25 to 35 min). Following this another LAMP run was conducted to determine the best dilution to be used as synthetic DNA for positive template in LAMP assay. The same fourfold serial dilution (1.0 ng/µL to 0.00098 ng/µL) of clean phylloxera DNA prepared previously was used as template to compare with one hundred thousand copies (10^5^) of synthetic DNA. The amount of phylloxera DNA was then calculated from the amplification time of 10^5^ copies of synthetic DNA.

## Supplementary information

Supplementary Information.

## Data Availability

GenBank, accession numbers MW292598 - MW292628.
