# Supplementary material for: A diagnostic LAMP assay for the destructive grapevine insect pest, phylloxera (Daktulosphaira vitifoliae)
Source: Sci Rep. 2020 Dec 4;10:21229. doi: 10.1038/s41598-020-77928-9 (PMC7718921; doi:10.1038/s41598-020-77928-9)
Supplement: Supplementary file 1 — Supplementary Information. [file 41598_2020_77928_MOESM1_ESM.docx]

**Supplementary Information**

**A diagnostic LAMP assay for the destructive grapevine insect pest, phylloxera (*Daktulosphaira vitifoliae*)**

Arati Agarwal ^1^, J. Paul Cunningham ^1, 2^, Isabel Valenzuela ^1^ & Mark J. Blacket ^1*^

^1^ Agriculture Victoria Research, AgriBio, 5 Ring Road. Bundoora, Victoria 3083, Australia.

^2^ School of Applied Systems Biology, La Trobe University, Bundoora, Victoria 3083, Australia.


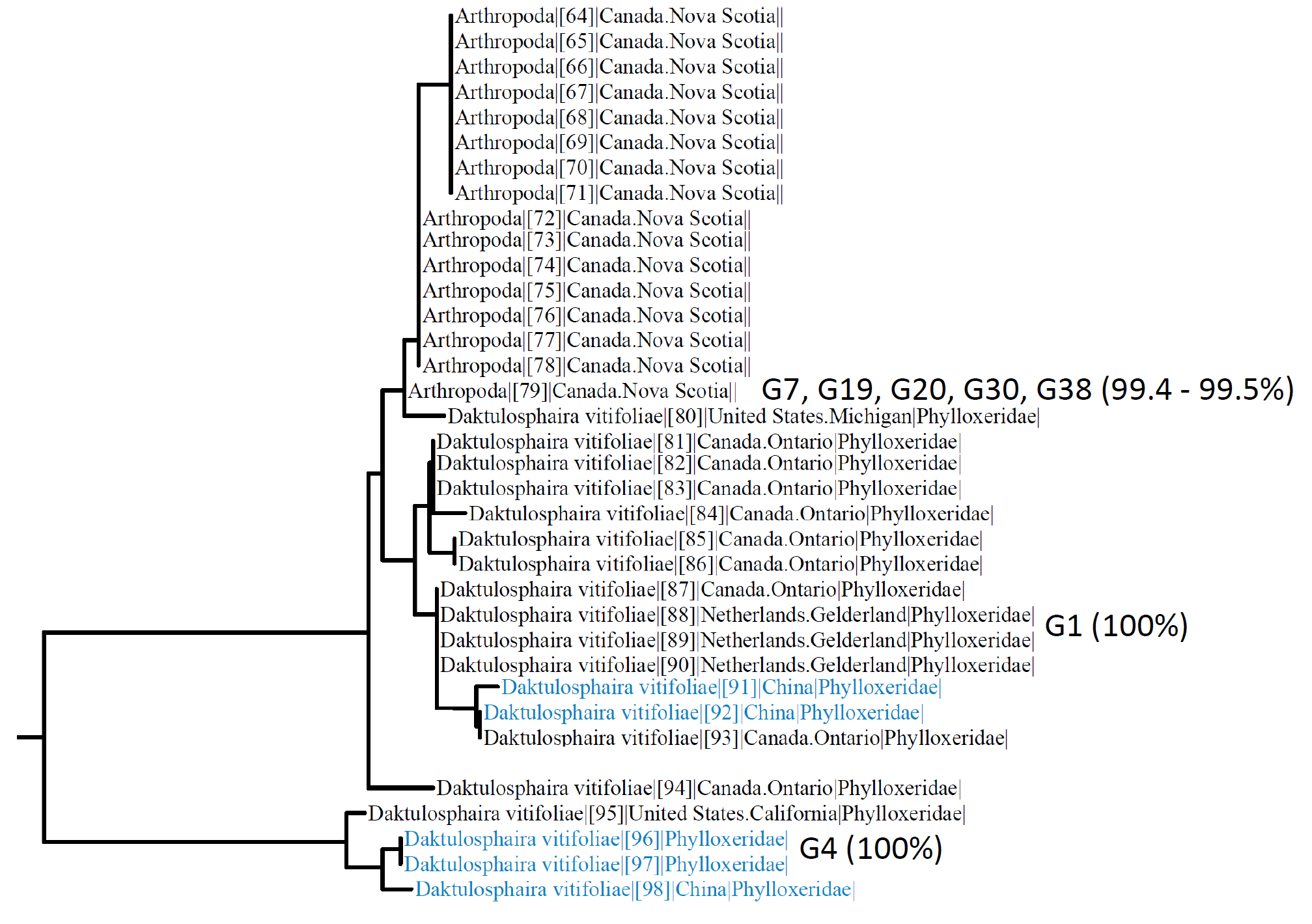


**Supplementary Figure 1.** Closest matches of Australian phylloxera strains with reference DNA barcode sequences on BOLD (accessed July 2020). Percentage match of each strain with closest BOLD reference sequence shown in brackets.

**Supplementary Table 1.** Target and non-target Aphidomorpha (Phylloxeroidea & Aphidoidea) used for LAMP primer design.

| **Species** | **Superfamily** | **Family** | **Reason for including species** | **Refererence** | **Accession number (BOLD)** |
| --- | --- | --- | --- | --- | --- |
| *Daktulosphaira vitifoliae* | Phylloxeroidea | Phylloxeridae | Phylloxera, the target species. | 1, 2 | 230 x sequences on BOLD |
| *Phylloxerina salicis* | Phylloxeroidea | Phylloxeridae | To account for Phylloxeroidea diversity, present in Australia. | 1 | GBMHH6688-15 |
| *Adelges normannianae* | Phylloxeroidea | Adelgidae | To account for Phylloxeroidea diversity, present in Australia. | 1 | GBMHH1826-13 |
| *Pineus pini* | Phylloxeroidea | Adelgidae | To account for Phylloxeroidea diversity, present in Australia. | 1 | GBMHH187-10 |
| *Aphis craccivora* | Aphidoidea | Aphididae | Aphid species known from grapevines (host). | 2 | ACEA141-14 |
| *Aphis fabae* | Aphidoidea | Aphididae | Aphid species known from grapevines (host). | 2 | ACEA050-14 |
| *Aphis gossypii* | Aphidoidea | Aphididae | Aphid species known from grapevines (host). | 2 | ACE1143-14 |
| *Aphis illinoisensis* | Aphidoidea | Aphididae | Aphid species known from grapevines (host). | 2 | RFBAE1197-10 |
| *Aphis hederae* | Aphidoidea | Aphididae | Aphid species known from grapevines (host). | 2 | ACEA062-14 |
| *Aulacorthum solani* | Aphidoidea | Aphididae | Aphid species known from grapevines (host). | 2 | ACEA131-14 |
| *Macrosiphum euphorbiae* | Aphidoidea | Aphididae | Aphid species known from grapevines (host). | 2 | ACEA250-14 |
| *Geoica wertheimae* | Aphidoidea | Aphididae | A close relative (not present on BOLD) is known from grapes. | 2 | RDBA251-05 |
| *Prociphilus caryae* | Aphidoidea | Aphididae | A close relative (not present on BOLD) is known from grapes. | 2 | BBHCN1096-10 |
| *Aploneura lentisci* | Aphidoidea | Aphididae | Root aphid present in Victoria, Australia. | 1, 2 | ACEA790-14 |

***References:***

1. Ratnasingham, S. & Hebert, P. D. N. BOLD: The barcode of life data system. Mol. Ecol. Notes 7, 355–364 (2007).

2. Blackman, R. L. & Eastop, V. F. Aphids on the World’s Crops: An Identification and Information Guide 2nd edn. (Wiley, London,

2000).
